# Spherical V_2_O_5_/C Cathode Materials Prepared by Spray Drying for High-Power Thermal Batteries

**DOI:** 10.3390/nano16130791

**Published:** 2026-06-24

**Authors:** Yaning Chang, Chuanyu Jin, Shaoming Qiao, Xianghua Zhang, Yujing Zhu, Yongxu Du

**Affiliations:** 1 College of Material Science and Engineering, Liaocheng University, Liaocheng 252000, China; yaninglcu@sina.com (Y.C.); zhangxianghua@lcu.edu.cn (X.Z.); zhuyujing@lcu.edu.cn (Y.Z.); duyongxu@lcu.edu.cn (Y.D.); 2 State Key Laboratory of Advanced Chemical Power Sources, Guizhou Meiling Power Sources Co., Ltd., Zunyi 563003, China

**Keywords:** V_2_O_5_, cathode materials, spray-drying, high-power thermal batteries

## Abstract

Commercial V_2_O_5_ powders typically exhibit a lamellar morphology with limited structural stability and sluggish electron/ion transport, which restricts their discharge performance in thermal batteries. This work aims to enhance the discharge performance of V_2_O_5_ cathodes by constructing a robust spherical architecture via a scalable spray drying strategy combined with carbon modification. The as-prepared V_2_O_5_/C cathode delivers a high initial discharge voltage of 2.45 V, a specific capacity of 261.06 mAh g^−1^, and an energy density of 591.05 Wh kg^−1^ at 0.1 A cm^−2^ and 500 °C (cut-off voltage of 1.9 V), outperforming those of commercial V_2_O_5_ cathodes. Pulse discharge tests and resistance evolution analyses further demonstrate enhanced voltage stability and reduced interfacial resistance. These improvements originate from the synergistic effect of the spherical architecture and conductive carbon network, which facilitates continuous electron/ion transport and reinforces structural integrity under high-temperature discharge conditions. This work provides a scalable design strategy for high-tap-density spherical V_2_O_5_ cathodes and offers insight into the coupling among morphology, conductivity, and stability in cathode materials for thermal battery.

## 1. Introduction

Thermal batteries are a class of high-temperature reserve batteries based on molten salt electrolytes [1,2,3]. They are activated within milliseconds by an internal heat source that melts the electrolyte, enabling ionic conductivities (>1 S·cm^−1^) at elevated temperatures (≥500 °C) and thereby delivering instantaneous high-power output [4,5,6]. In contrast to conventional chemical batteries, thermal batteries remain inert under natural conditions, while offering ultralong storage life (>20 years), rapid activation, a wide operating temperature range, and high reliability, making them indispensable for missile guidance, spacecraft emergency power supply, and deep-sea exploration in extreme environments [7,8,9].

The discharge performance of thermal batteries largely depends on the cathode materials [10,11,12,13]. Commercial cathode materials (FeS_2_, NiS_2_, and CoS_2_) are typically synthesized by the mechanical mixing and high-temperature solid-state method [14]. The precursor powders are ball-milled and subsequently sintered at >500 °C to achieve phase formation and crystallization [15]. Although the solid-state method is simple and cost-effective, the broad particle size distributions and severe agglomeration limit interfacial contact between active materials and electrolyte, thereby slowing interfacial reaction kinetics [16,17]. High-temperature sintering also promotes excessive grain growth, undermining structural stability. The limited morphological control leads to low tap density, which is unfavorable for powder pressing in cathode electrode of thermal batteries. The hydrothermal method has been explored to synthesize uniform, nanoscale cathode materials, which partially mitigates the particle growth and dispersion limitations associated with the solid-state route [18,19,20]. However, the prolonged reaction times (typically 12–48 h) and high equipment costs hinder large-scale production [21]. The process generates substantial wastewater, posing environmental concerns. Although transition metal sulfides are suitable for long-life thermal batteries, their relatively low open-circuit voltage makes it difficult to meet the requirements of high-power thermal batteries. To satisfy the power-density requirements of extreme environment applications, halide cathode materials (NiCl_2_ and FeF_3_) have attracted increasing attention owing to their high redox potentials [8]. The mainstream synthesis method for these materials is based on the thermal decomposition of commercially available hydrated crystalline precursors (e.g., NiCl_2_·6H_2_O, FeF_3_·3H_2_O) [22,23]. The process involves controlled heating rate (usually 5–10 °C min^−1^) to gradually remove crystallization water (200–300 °C) in an inert atmosphere, followed by further heating to 500–800 °C to induce sublimation–recrystallization, yielding high-purity amorphous or microcrystalline NiCl_2_ and FeF_3_ cathode materials. The advantages of the method include the availability of raw materials and a simple process route. However, high-temperature dehydration may induce localized overheating, leading to impurity formation (NiO, Fe_3_O_4_). Furthermore, sublimation–recrystallization generates large plate-like particles with low tap density.

**Figure 1:**
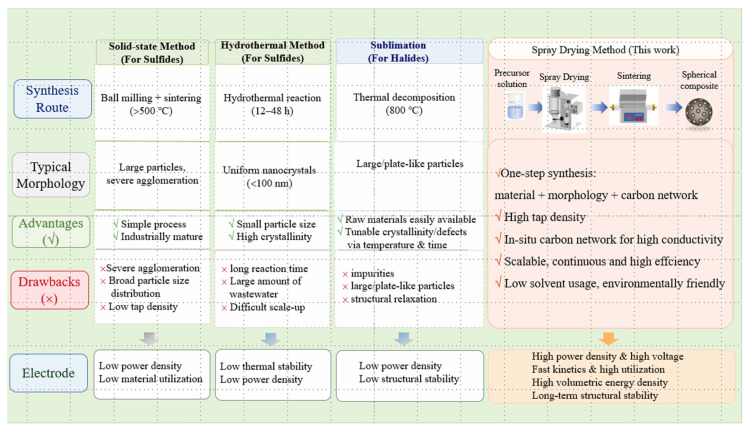
Comparison of synthesis methods for cathode materials of thermal battery.

Recent studies on MOF-based electrode materials have demonstrated that rational structural engineering plays a crucial role in optimizing electrochemical performance. The enhanced ion/electron transport achieved through tailored porous architectures highlights the importance of morphology control in electrode design [24,25]. As shown in Figure 1, spray drying has attracted increasing attention for electrode powders owing to its versatility in material design [26,27]. This technique atomizes precursor solutions into micron-sized droplets, which are rapidly dried in a high-temperature airflow, thereby enabling material synthesis, morphology control, and microstructural optimization in a single step [28]. Spherical architectures with large surface areas can be obtained by tailoring precursor concentration, atomization conditions, and drying temperature, thereby providing abundant electrochemically active sites [29]. The process also enables the formation of homogeneous carbon networks, which improve electronic conductivity and structural integrity. In addition, the high tap density of spray-dried powders facilitates electrode compaction and contributes to high volumetric energy density.

Vanadium pentoxide (V_2_O_5_), a low-cost and abundant layered transition metal oxide, has a theoretical capacity of up to 440 mAh g^−1^, and a moderate redox potential (~3.0 V vs. Li/Li^+^), showing unique advantages in high-power thermal battery [30]. The cost of V_2_O_5_ is significantly lower compared to traditional sulfides (such as FeS_2_) or novel nickel-based materials (such as NiCl_2_), further enhancing its commercialization potential [31]. However, commercial V_2_O_5_ powders are produced through industrial wet metallurgy processes, which leads to their flaky or blocky morphology and low tap density and their low electronic conductivity results in pronounced polarization during high-rate discharge [32]. Studies combining spherical structural engineering and carbon network construction in a scalable fabrication process remain scarce for thermal battery applications. Therefore, developing high-tap-density spherical V_2_O_5_/C cathodes with enhanced conductivity and structural stability represents an important yet insufficiently explored research direction.

In this study, commercial V_2_O_5_ powders were used as the raw material, and spray drying technology was employed to fabricate spherical V_2_O_5_ particles with regular morphology, enhancing the formability and tap density, thus improving its power output characteristics in thermal batteries. To further improve the conductivity and structural stability of V_2_O_5_ cathode material, 2-Methylimidazole was introduced as a structure modifier and carbon source. 2-Methylimidazole improves precursor cohesion and promotes spherical particle formation, while its carbonization during heat treatment generates a conductive carbon network uniformly distributed throughout the particles. This work aims to reveal the underlying mechanisms of multi-scale structure regulation and performance enhancement in V_2_O_5_ cathode materials prepared via the spray drying method combined with carbon modification, thereby advancing the development and engineering of cathode materials in high-power thermal batteries through systematic characterization of the phase structure, morphological evolution, and electrochemical performance of the products under various preparation parameters.

## 2. Materials and Methods

### 2.1. Materials

All chemicals used in this study were of analytical grade and employed without further purification. Commercial V_2_O_5_ (Shanghai Macklin Biochemical Co., Ltd. Shanghai, China) and 2-Methylimidazole (Shanghai Aladdin Biochemical Technology Co., Ltd. Shanghai, China) were utilized as received in this paper.

### 2.2. Synthesis of V_2_O_5_ Cathode Materials

**Figure 2:**
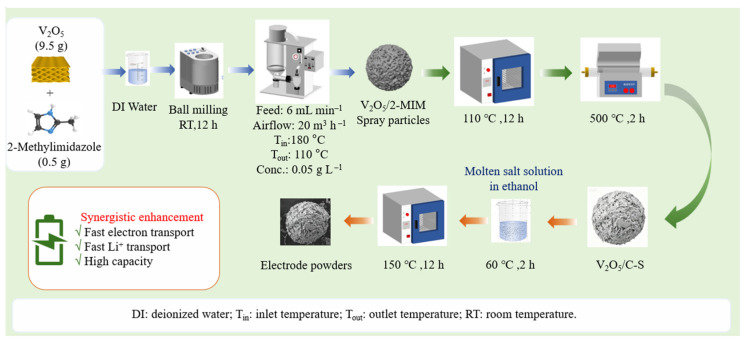
Schematic diagram of spray drying method for preparing V_2_O_5_ composites.

The experimental procedure is shown in Figure 2. First, commercial vanadium pentoxide (V_2_O_5_) and 2-Methylimidazole were added to deionized water in a mass ratio of 9.5:0.5. The mixtures were continuously stirred using a ball mill for 6 h to form a uniform precursor emulsion, ensuring no significant precipitation or stratification. The prepared emulsion was then rapidly dried using spray drying equipment with the following parameters: a feed rate of 6 mL min^−1^, an airflow rate of 20 m^3^ h^−1^, an inlet temperature of 180 °C, an outlet temperature controlled at 110 °C, and a precursor solution concentration maintained at 0.05 g L^−1^. The V_2_O_5_/2-Methylimidazole spray particles were collected and placed in a vacuum drying oven at 110 °C for 12 h to thoroughly remove residual moisture from both internal and surface areas, yielding dry spray particle precursors. Finally, the dried particles were sintered in a tube furnace at 500 °C for 2 h under nitrogen protection with a controlled heating rate of 5 °C min^−1^ to ensure complete pyrolysis of the carbon source and promote the formation and stabilization of the V_2_O_5_ crystal phase. The sintered material was cooled to room temperature to obtain the final V_2_O_5_ composite cathode materials (denoted as V_2_O_5_/C-S). To investigate the effect of 2-Methylimidazole-derived carbon on electrochemical performance, pure V_2_O_5_ was prepared using the spray drying method and designated as V_2_O_5_-S. And pure V_2_O_5_ was designated as V_2_O_5_-R.

To construct multi-scale lithium-ion transport channels, 0.2 g of ternary molten salt (9.6 wt.% LiF-22 wt.% LiCl-68.4 wt.% LiBr; melting point 436 °C) was dissolved in 30 mL of ethanol at 50 °C under stirring. Then, 0.8 g of the as-prepared cathode material was gradually added to the solution along with the stir at 60 °C for 2 h. The product was dried at 150 °C for 12 h and subsequently cooled in a glove box for further using.

### 2.3. Characterization

Crystal structure was analyzed through X-ray diffraction (XRD, Siemens D500, Siemens AG, Munich, Germany) using Cu Kα radiation (λ = 1.5418 Å) over a 2θ range of 10–90°. Morphology and elemental distribution were characterized through field emission scanning electron microscopy (FE-SEM, Zeiss Sigma 500, Carl Zeiss AG, Oberkochen, Germany) coupled with energy-dispersive X-ray spectroscopy (EDS, Oxford X-Max 50, Oxford Instruments plc, Abingdon, UK). Specific surface area and pore structure were determined from nitrogen adsorption–desorption isotherms at 77 K using the Brunauer–Emmett–Teller (BET) method (ASAP 2460, Micromeritics Instrument Corp., Norcross, GA, USA). The tap density was measured using a tap density analyzer (HM-ZS1, Shandong Hengmei Electronic Technology Co., Ltd., Weifang, China). Thermal stability was evaluated through thermogravimetric analysis (TGA, NETZSCH STA 449F5, NETZSCH-Gerätebau GmbH, Selb, Germany) under air atmosphere with a heating rate of 10 °C min^−1^ from 25 °C to 800 °C. Surface chemistry was examined through X-ray photoelectron spectroscopy (XPS, ESCALAB 250Xi, Thermo Fisher Scientific, Waltham, MA, USA). Additional microstructural observation was performed using an ultra-depth three-dimensional microscope (KEYENCE VHX-1000, Keyence Corporation, Osaka, Japan).

### 2.4. Assembly Single Cell and Discharge Measurements

A single thermal battery cell comprises a cathode electrode, a separator layer (composed of 9.6 wt.% LiF–22 wt.% LiCl–68.4 wt.% LiBr, melting point 436 °C, and 50 wt.% MgO, pressed into 20 mm pellets under 270 MPa), and an anode (20 mm LiB alloy, thickness 0.6 mm, provided by Hunan Ruilin New Energy Technology Co., Ltd., Changsha, China). For cell assembly, 0.20 g of cathode powder and 0.40 g of solid electrolyte were sequentially layered in a die and pressed at 270 MPa to form a 20 mm diameter pellet. All procedures were conducted in a glove box with H_2_O and O_2_ levels below 0.1 ppm. The assembled cells were thermally activated using a custom-designed heating system. Discharge performance (voltage and specific capacity) was evaluated using an electrochemical workstation (Gamry Interface 1010E, Gamry Instruments, Warminster, PA, USA and Wuhan Corrtest CS1350, Wuhan, China) and a programmable electronic load (ITECH IT8500 plus, ITECH Electronics Co., Ltd., Nanjing, China) in Figure S1. Specific capacity, specific energy, and pulse resistance were calculated using Equations (S1)–(S3) in the Supporting Information.

## 3. Results

**Figure 3:**
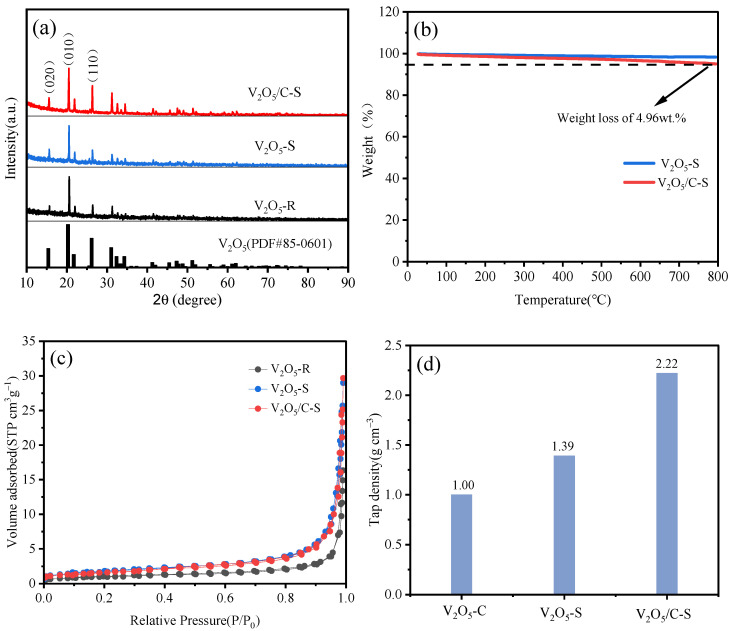
(**a**) XRD, (**b**) TG, (**c**) BET and (**d**) tap density of V_2_O_5_-R, V_2_O_5_-S, and V_2_O_5_/C-S cathode powders.

Figure 3a presents the X-ray diffraction (XRD) patterns of commercial (raw materials) V_2_O_5_ (V_2_O_5_-R), spray-dried V_2_O_5_ (V_2_O_5_-S), and spray-dried carbon-modified V_2_O_5_ composite (V_2_O_5_/C-S). The diffraction peaks at 15.37°, 20.29°, and 26.15° correspond to the (020), (010), and (110) planes of V_2_O_5_ (PDF#85-0601). The diffraction peaks of all samples are well matched with the reference data, exhibiting sharp profiles and high intensities, which indicate good crystallinity and high phase purity of the obtained materials. Notably, V_2_O_5_/C-S powders show neither peak shifts nor additional peaks, confirming that carbon incorporation does not induce lattice distortion or secondary phase formation, thereby preserving the integrity of the V_2_O_5_ structure. Figure 3b displays thermogravimetric (TG) curves of V_2_O_5_-S and V_2_O_5_/C-S. V_2_O_5_-S exhibits almost no weight loss in the range of 25–800 °C and demonstrates excellent thermal stability. In contrast, V_2_O_5_/C-S shows a weight loss of approximately 4.96 wt.% within the same temperature window, which can be attributed to the oxidative decomposition of amorphous carbon species in air. The pyrolytic carbon content in V_2_O_5_/C-S composite is approximately 4.96 wt.%. Figure 3c shows the N_2_ adsorption–desorption isotherms and the corresponding Brunauer–Emmett–Teller (BET) surface areas of V_2_O_5_-R, V_2_O_5_-S, and V_2_O_5_/C-S. All samples exhibit typical type-IV isotherms, suggesting the presence of mesoporous structures. Specifically, BET surface area of V_2_O_5_-S reaches 6.71 m^2^ g^−1^, which is ~74.7% higher than that of V_2_O_5_-R (3.84 m^2^ g^−1^), indicating that spray drying facilitates the formation of a looser particle packing structure with more exposed active surfaces. The surface area of V_2_O_5_/C-S powders decreases slightly to 5.42 m^2^ g^−1^ (18.9% lower than that of V_2_O_5_-S powders), likely due to partial particle aggregation and the presence of a carbon coating layer. The cathode electrode of thermal batteries is fabricated by the powder compaction process, and the tap density serves as a critical performance parameter. A low tap density can lead to insufficient densification of the pressed electrode, resulting in structural degradation and hindered charge transport pathways. These could reduce the utilization efficiency of active materials and may even cause catastrophic battery failure in severe cases. Figure 3d shows that commercial V_2_O_5_ exhibits the lowest tap density of approximately 1.00 g cm^−3^. V_2_O_5_-S shows an increased tap density of about 1.39 g cm^−3^ after simple spray drying treatment, which can be attributed to the agglomeration of particles and optimized particle size distribution during the spray drying process, leading to improved packing efficiency and space-filling ability. The conventional carbon incorporation generally reduces tap density due to the introduction of low-density carbon phases. Remarkably, the spray drying combined with pyrolytic carbon modification yields V_2_O_5_/C-S with the highest tap density (2.22 g cm^−3^), indicating that the synergistic structural densification and uniform carbon modification can enhance electronic conductivity while maintaining efficient particle packing.

**Figure 4:**
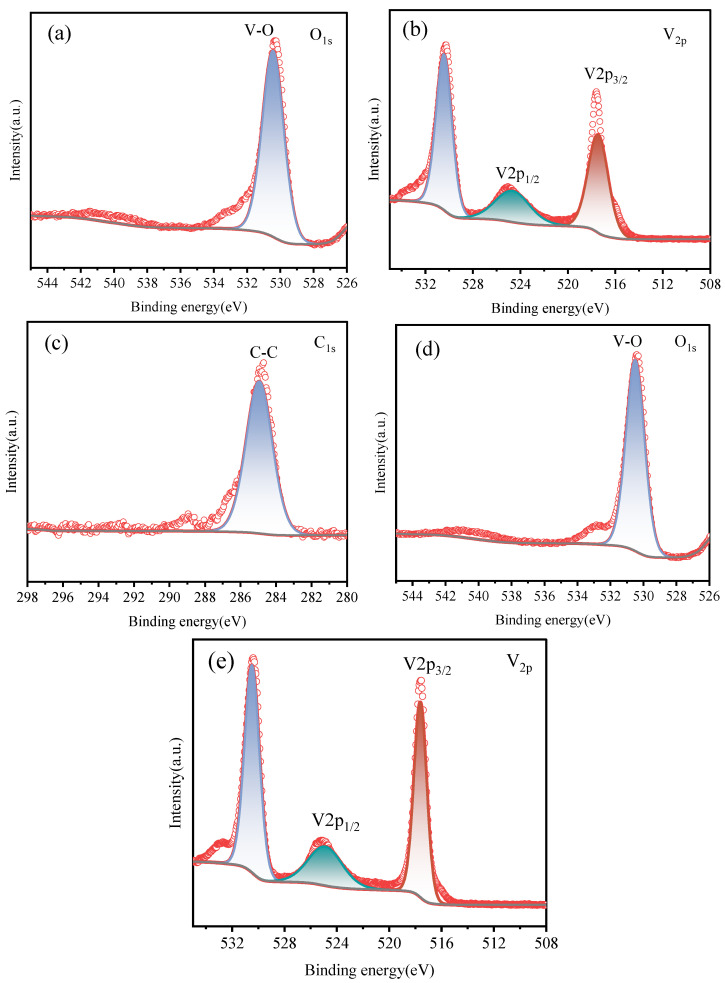
XPS of (**a**,**b**) V_2_O_5_-S and (**c**–**e**) V_2_O_5_/C-S cathode powders.

X-ray photoelectron spectroscopy (XPS) is employed to elucidate the chemical states of elements in the V_2_O_5_-S and V_2_O_5_/C-S cathode materials. As shown in Figure 4a, the O 1s spectrum of the V_2_O_5_-S sample exhibits a characteristic peak at 530.2 eV, corresponding to V–O bonds, confirming the formation of vanadium oxide framework. Figure 4b presents the V 2p high-resolution spectrum, which can be deconvoluted into two peaks located at 517.65 eV (2p_3/2_) and 525.75 eV (2p_1/2_), indicating that vanadium predominantly exists in the +5 oxidation state. For the V_2_O_5_/C-S cathode, Figure 4c shows the C 1s spectrum with a prominent peak at 284.47 eV, attributed to C–C bonds derived from carbonization of the organic precursor during synthesis. Figure 4d presents the O 1s spectrum, which also shows a peak at approximately 530.35 eV corresponding to V–O bonds, indicating that the introduction of carbon does not alter the fundamental vanadium oxide structure. Finally, Figure 4e displays the V 2p spectrum of the V_2_O_5_/C-S sample, which is well consistent with that of pristine V_2_O_5_, confirming that vanadium remains in the +5 oxidation state after carbon modification.

**Figure 5:**
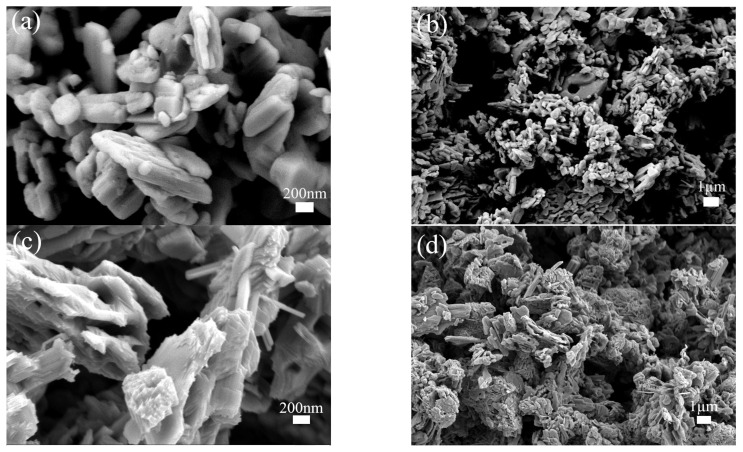
SEM of (**a**,**b**) V_2_O_5_-R and (**c**,**d**) V_2_O_5_-S powders.

Figure 5a,b present the typical morphology of commercial V_2_O_5_ powders, which consists of irregularly aggregated short rod-like and plate-like particles forming a relatively dense structure. In contrast, Figure 5c,d show the morphology of V_2_O_5_-S obtained after spray treatment, exhibiting more uniform distribution with well-defined plate-like and rod-like structures. It can also be observed that the spray drying alone is insufficient to induce the formation of spherical secondary particles when using pure V_2_O_5_ as the precursor without any binder or additional additives. The morphology remains anisotropic rather than being fully spheroidized in the spray drying process.

**Figure 6:**
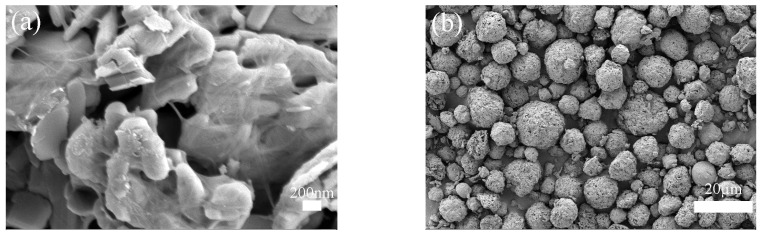

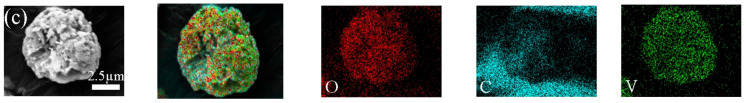
(**a**,**b**) SEM and (**c**) EDS of V_2_O_5_/C-S cathode materials.

Figure 6 presents morphological features and elemental distribution of V_2_O_5_/C-S characterized by SEM and energy-dispersive X-ray spectroscopy (EDS). Figure 6a shows that V_2_O_5_/C-S powders exhibit a complex layered or sheet-like structure, with particles interconnected through a carbon network framework. The sample evolves into uniform spherical or quasi-spherical particles in Figure 6b, which is favorable for enhancing packing density and structural stability. The EDS indicates that O and V are homogeneously distributed within the particles with a high degree of overlap in Figure 6c, while the network structure is identified as pyrolytic carbon. The C element is predominantly enriched at the particle surfaces and edges, which may originate from the decomposition or reaction of 2-Methylimidazole during the sintering process. The organic ligand serves as both a structure-directing agent and a carbon precursor. It promotes the formation of uniform spherical precursor particles through coordination and improved droplet stability during the spray drying process. The organic ligand is converted into an amorphous carbon network that is uniformly distributed within the secondary particles upon sintering, enhancing electronic conductivity and contributing to the structural integrity of the spherical V_2_O_5_/C cathodes. Collectively, these results demonstrate that V_2_O_5_/C-S forms a composite material with uniform spherical morphology, while maintaining a homogeneous spatial distribution of V_2_O_5_ and C elements.

**Figure 7:**
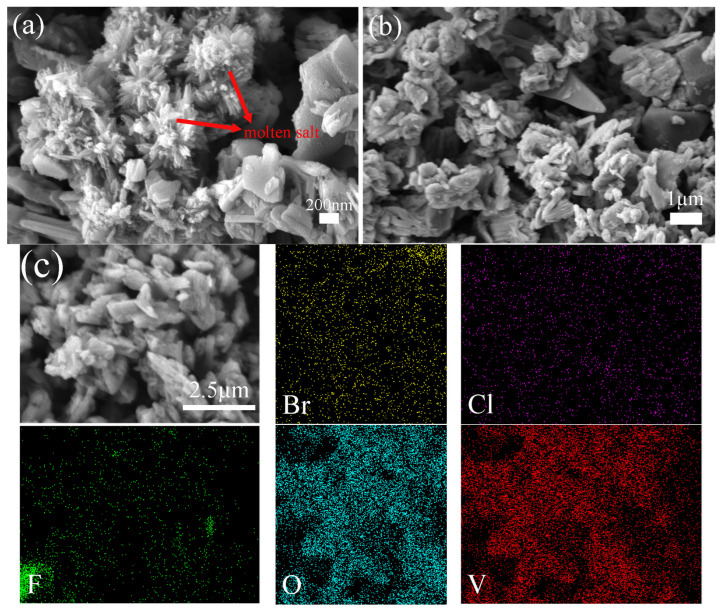
(**a**,**b**) SEM and (**c**)EDS of V_2_O_5_-S/molten salt cathode electrode powders.

The mixing homogeneity between molten salt electrolytes and cathode active materials plays a critical role in determining ionic transport performance in cathode electrodes. Figure 7a,b show SEM images of V_2_O_5_-S/molten salt cathode electrode powders. V_2_O_5_-S retains the original layered structure, but heterogeneous deposition of molten salt appears on its surface after molten salt treatment. The EDS elemental distribution map reveals the distribution of Br, Cl, F, O, and V elements in V_2_O_5_-S after molten salt impregnation. The results indicate that the V/O elements maintain a high degree of spatial coupling, while the distribution of Br, Cl, and F elements is uneven, with enrichment primarily occurring at the edges of aggregates or cracks on the layered surfaces. This suggests that the molten salt did not effectively adhere to the V_2_O_5_-S surface and failed to significantly improve ion transport properties.

**Figure 8:**
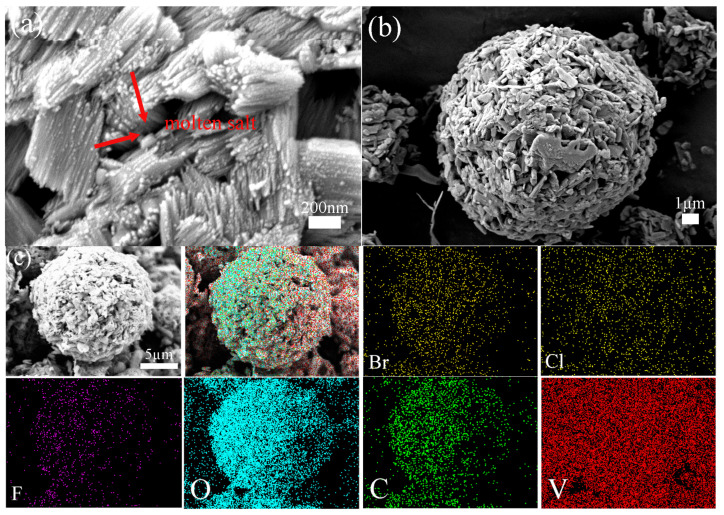
(**a**,**b**) SEM and (**c**) EDS of V_2_O_5_/C-S/molten salt cathode electrode powders.

Figure 8 presents SEM images of V_2_O_5_/C-S after molten salt impregnation. The molten salt in V_2_O_5_/C-S is more uniform distribution than that in V_2_O_5_-S powders, with finer particles and no evident agglomeration. V_2_O_5_/C-S exhibits a structure formed by stacked nanosheets at the nanoscale (200 nm), and it shows spherical particles with rough surfaces and distinct porosity at the microscale (1 µm). The EDS elemental mapping further reveals the spatial distribution of Br, C, Cl, F, O, and V within the composite. The results indicate that V and O, as the principal constituents of V_2_O_5_, are highly consistent with both spherical and sheet-like structures, confirming V_2_O_5_ as the primary framework of the material. Meanwhile, C is uniformly distributed and partially overlaps with V and O, suggesting homogeneous and effective carbon incorporation. Additionally, Br, Cl, and F exhibit significant overlap and are mainly concentrated in the spherical particle regions, confirming the uniform dispersion of molten salts within the V_2_O_5_/C-S cathode electrode powders.

**Figure 9:**
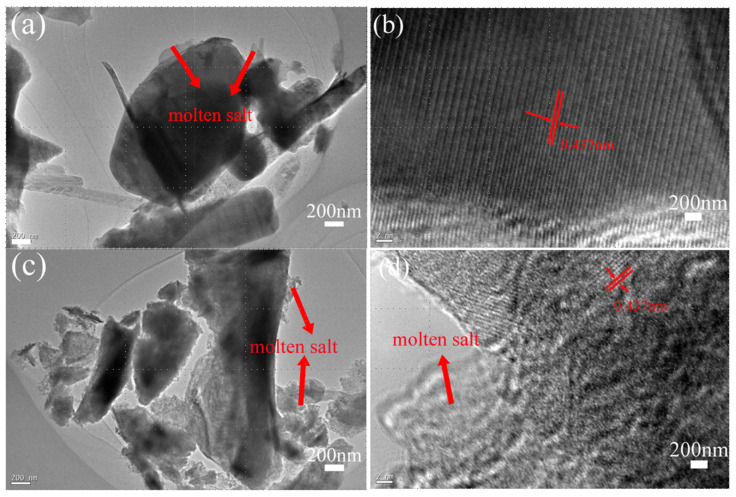
TEM of (**a**,**b**) V_2_O_5_-S/molten salt and (**c**,**d**) V_2_O_5_/C-S/molten salt.

Figure 9a,b show that a small amount of molten salt is attached to the surface of the V_2_O_5_-S nanosheets. Clear lattice fringes with an interplanar spacing of 0.437 nm can be attributed to the characteristic crystal planes (001) of V_2_O_5_, indicating insufficient molten salt coverage on the active material surface. The contact area between V_2_O_5_ and the molten salt is limited, which could restrict the formation of efficient ionic transport pathways. Figure 9c,d show the TEM and HRTEM images of V_2_O_5_/C-S after mixing with molten salt. A large amount of molten salt is uniformly distributed on the surface of the V_2_O_5_ nanosheets and infiltrates the inter-nanosheet voids, forming a continuous and intimate interfacial contact layer. The lattice fringes of V_2_O_5_ become significantly weaker or are partially obscured in HRTEM, suggesting the formation of a highly covered molten salt coating layer on the active material surface. Such a homogeneous molten salt network facilitates the construction of continuous Li^+^ transport pathways, thereby promoting interfacial ion migration and enhancing the electrochemical reaction kinetics of the electrode.

**Figure 10:**
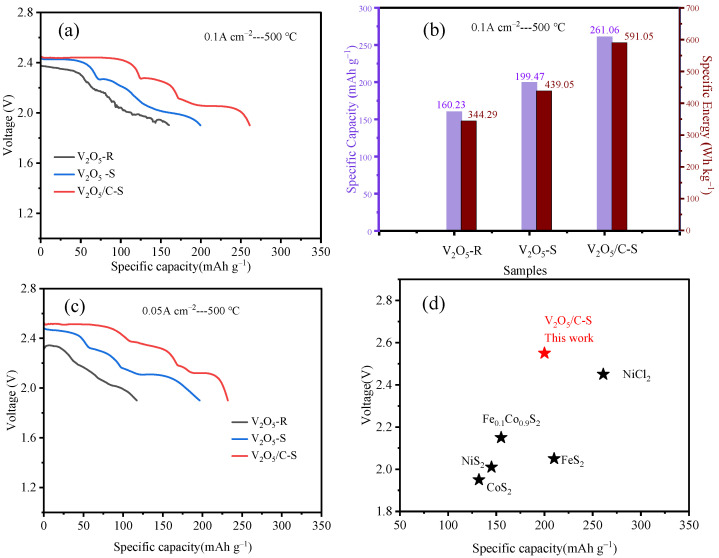
Discharge performance of V_2_O_5_-R, V_2_O_5_-S, and V_2_O_5_/C-S cathode at (**a**,**b**) 0.1 A cm^−2^, (**c**) 0.05 A cm^−2^ and (**d**) comparison of discharge performance with 0.1 A cm^−2^ and a cut-off voltage of 1.9 V [3,14,33,34].

To systematically investigate the correlation between spherical structure and discharge performance, discharge tests are conducted at 0.1 A cm^−2^ and 0.05 A cm^−2^. The initial discharge voltage of V_2_O_5_/C-S cathode reaches 2.45 V at 500 °C and 0.1 A cm^−2^ in Figure 10a, which is significantly higher than 2.37 V of V_2_O_5_-R cathode and 2.43 V of V_2_O_5_-S cathode. The V_2_O_5_/C-S cathode exhibits two distinct discharge plateaus at 2.43 V and 2.05 V, whereas neither V_2_O_5_-R nor V_2_O_5_-S displayed such features. The discharge profile of V_2_O_5_/C-S cathode remains more stable prior to the cut-off voltage of 1.9 V, indicating a reduced polarization effect. These results demonstrate that spray drying not only enables the formation of spherical particles but also facilitates the construction of continuous electron pathways and ion diffusion channels, thereby mitigating polarization and enhancing discharge voltage, plateau stability, and specific capacity. Both the specific capacity and specific energy of V_2_O_5_/C-S cathode are significantly improved compared with the other samples in Figure 10b. V_2_O_5_/C-S cathode delivers a specific capacity of 261.06 mAh g^−1^ at a cut-off voltage of 1.9 V, much higher than 160.23 mAh g^−1^ of the V_2_O_5_-R cathode and 199.47 mAh g^−1^ of the V_2_O_5_-S cathode. The specific energy of V_2_O_5_/C-S cathode reaches 591.05 Wh kg^−1^, which is also markedly superior to 344.29 Wh kg^−1^ of V_2_O_5_-R cathode and 439.05 Wh kg^−1^ of V_2_O_5_-S cathode. To further evaluate the structural stability of the spherical morphology under prolonged exposure to high temperatures and molten salts, discharge tests are conducted at a low current density of 0.05 A cm^−2^ (longer discharge durations, Figure 10c). The results reveal that the V_2_O_5_/C-S cathode still exhibits outstanding discharge performance, maintaining a specific capacity of 232.02 mAh g^−1^ and a specific energy of 543.5 Wh kg^−1^. Spherical V_2_O_5_/C-S architecture uniquely integrates structural robustness, continuous conductive pathways, and scalable manufacturability. So V_2_O_5_/C-S prepared in this work exhibits superior electrochemical performance compared with transition metal sulfide cathodes at a high-voltage platform (with a cut-off voltage of 1.9 V) in Figure 10d. A V_2_O_5_/C-S cathode prepared via spray drying demonstrates superior discharge performance, characterized by higher discharge voltages, more stable plateaus, and enhanced specific capacity and energy density, highlighting its significant potential as a cathode material for thermal batteries [33,34].

**Figure 11:**
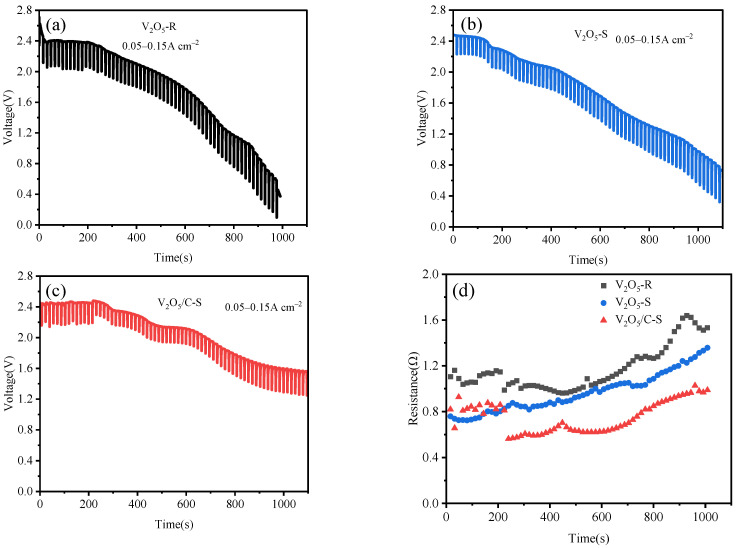
Pulse discharge curves and of (**a**) V_2_O_5_-R, (**b**) V_2_O_5_-S, and (**c**) V_2_O_5_/C-S cathode and (**d**) pulse resistance.

Figure 11 presents the pulse discharge curves and resistance evolution of V_2_O_5_-R, V_2_O_5_-S, and V_2_O_5_/C-S cathodes at 500 °C under current densities alternating between 0.05 A cm^−2^ (15 s) and 0.15 A cm^−2^ (1 s). All samples exhibit typical stepped voltage responses, indicating the periodic polarization and depolarization processes during pulse loading. However, distinct differences in voltage stability and polarization behavior can be observed among the three cathodes. The V_2_O_5_/C-S cathode maintains a higher and more stable discharge voltage throughout the pulse cycling, with low voltage fluctuations, demonstrating superior voltage stability and lower polarization. In contrast, V_2_O_5_-S cathode shows a relatively faster voltage decay, while V_2_O_5_-R cathode exhibits pronounced voltage drops and larger oscillations, suggesting that plate-like structure is insufficient to simultaneously ensure interfacial stability. The pulse resistance of all cathodes gradually increases with discharge time due to the accumulation of reaction products and elongation of ion diffusion paths. Notably, the V_2_O_5_/C-S cathode exhibits the lowest and most stable resistance throughout the entire process, confirming its improved interfacial charge transfer and structural integrity. The synergistic effects of carbon modification and the spherical architecture derived from spray drying significantly enhance electron/ion transport continuity, suppress polarization, and ensure stable high-power output. These results demonstrate that the V_2_O_5_/C-S cathode possesses high electrochemical kinetics and voltage retention, making it a promising candidate for thermal batteries.

**Figure 12:**
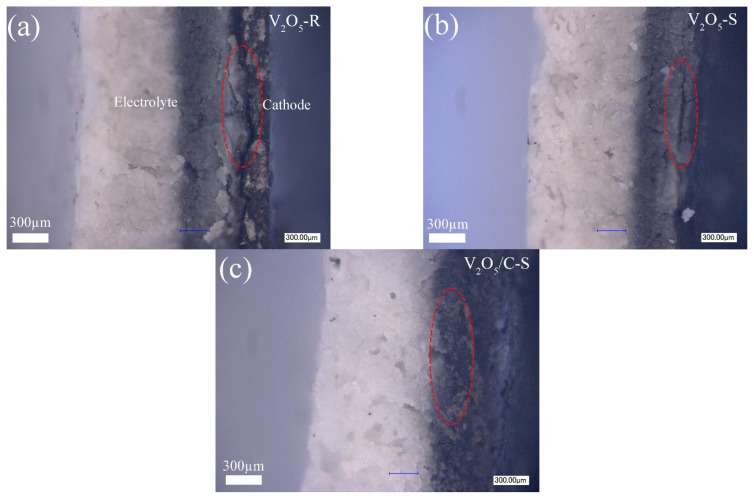
Interface and electrode changes in single cell after discharge test: (**a**) pure V_2_O_5_-R; (**b**) V_2_O_5_-S; (**c**) V_2_O_5_/C-S.

Figure 12 presents the optical microscopy images of the cathode/molten salt electrolyte interface after discharge. A pronounced interpenetration phenomenon is observed between the V_2_O_5_-R cathode and molten salt electrolyte in Figure 12a, resulting in a blurred interface boundary. In addition, obvious cracks and structural degradation are present within the cathode layer, indicating poor structural stability during discharge and the potential deterioration of interfacial transport pathways. After particle refinement and spray drying treatment, the V_2_O_5_-S cathode (Figure 12b) exhibits a significantly reduced degree of cathode/electrolyte interpenetration, accompanied by a clearer interface boundary and a stable cathode structure. The V_2_O_5_/C-S cathode (Figure 12c) maintains a distinct and intact cathode/electrolyte interface after discharge, with no obvious interpenetration or erosion caused by molten salt. Meanwhile, the cathode layer remains structurally intact and compact without noticeable cracking or collapse, demonstrating superior structural stability. The result could be attributed to the pyrolytic carbon-assisted spherical architecture, which not only promotes homogeneous molten salt distribution but also enhances structural robustness among particles, thereby effectively suppressing interfacial degradation and cathode cracking during discharge and improve discharge performance.

## 4. Conclusions

In this work, spherical V_2_O_5_/C cathode materials with high tap density were successfully synthesized via spray drying technology, and their electrochemical behavior in thermal batteries was systematically investigated. The spherical V_2_O_5_/C cathode exhibits a high initial discharge voltage of 2.45 V, stable voltage platforms, and enhanced specific capacity of 261.06 mAh g^−1^ and energy density of 591.05 Wh kg^−1^ at 0.1 A cm^−2^ and 500 °C (cut-off voltage of 1.9 V). The improved electrochemical performance is attributed to the spherical architecture, which provides continuous electron/ion transport pathways and alleviates polarization during discharge. Pulse discharge and resistance evolution analyses further confirm enhanced voltage stability and reduced interfacial resistance, indicating improved suitability for high-power thermal battery applications. This study demonstrates that a scalable spray drying strategy combined with carbon modification effectively addresses the intrinsic limitations of commercial V_2_O_5_, including its lamellar morphology, sluggish charge transport, and structural instability. The engineered spherical architecture, together with the conductive carbon network, provides continuous electron/ion transport pathways and enhances interfacial stability under high-temperature discharge conditions. These results suggest a practical and scalable design route for constructing high-performance V_2_O_5_-based cathode materials for thermal batteries.

Although the promising discharge performance is demonstrated in this work, several challenges remain for spherical V_2_O_5_/C cathodes, including further enhancement of electronic conductivity, stabilization of the cathode/electrolyte interface under high-temperature conditions, and optimization of structural integrity during discharge. Future efforts may focus on advanced carbon network design, interface engineering, and scalable manufacturing strategies to further improve the power output and reliability of thermal batteries.

## Data Availability

The original contributions presented in this study are included in the article/Supplementary Materials. Further inquiries can be directed to the corresponding author.

## Supplementary Materials

The following supporting information can be downloaded at: https://www.mdpi.com/article/10.3390/nano16130791/s1, Figure S1: Images of testing system of single thermal battery; Table S1: Comparison: Thermal batteries vs. Conventional lithium-ion batteries title.
