# Supplementary material for: Spherical V2O5/C Cathode Materials Prepared by Spray Drying for High-Power Thermal Batteries
Source: Nanomaterials (Basel). 2026 Jun 24;16(13):791. doi: 10.3390/nano16130791 (PMC13362719; doi:10.3390/nano16130791)
Supplement: Supplementary file 1 [file nanomaterials-16-00791-s001.zip › nanomaterials-4392042-supplementary.pdf]

Spherical V_2O_5/C cathode materials prepared by spray drying for high-power thermal batteries

Yaning Chang[1], Chuanyu Jin[1]*, Shaoming Qiao[2]*, Xianghua Zhang[1],
Yujing Zhu[1], Yongxu Du[1],
[1] College of Material Science and Engineering, Liaocheng University, Liaocheng 252000, China
[2] State Key Laboratory of Advanced Chemical Power Sources, Guizhou Meiling Power Sources Co. Ltd., Zunyi, Guizhou 563003, China
*Corresponding e-mail address: jinchuanyu@lcu.edu.cn (C. Jin), Qiaosmml@163.com (S. Qiao)

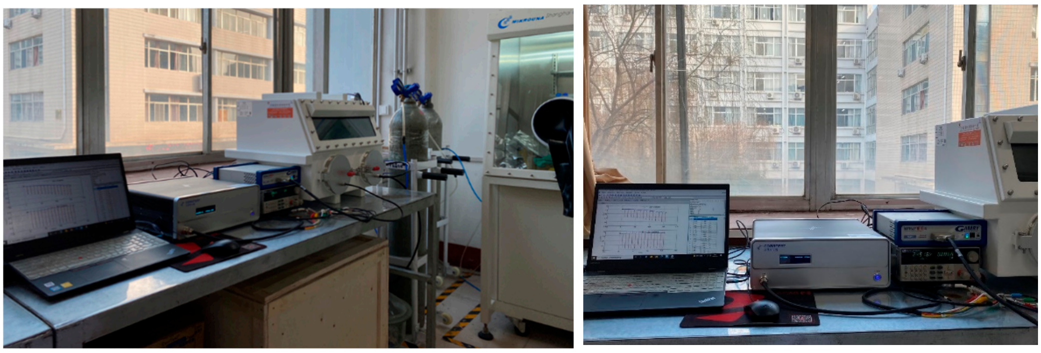

Figure S1. Images of testing system of single thermal battery
(the heating system is placed in the small glove box with Ar)

The following equations are used for calculating the specific capacity, specific energy, and pulse resistance, respectively.

$$P=It/3.6m \qquad (S1)$$

$$\varepsilon=\frac{\int UI\mathrm{d}t}{m} \qquad (S2)$$

$$R=(U_1-U_2)/(I_2-I_1) \qquad (S3)$$

P---specific capacity (mAh g^{-1})
ε---energy power density (Wh kg^{-1})
U---discharge voltage (V)
I---discharge current (A, $I=\pi r^2$ * 0.1 A cm^{-2}=3.14* $(0.1\ cm)^2$*0.1 A cm^{-2}=0.314 A)
t---discharge time (s)
m---mass of cathode materials
3.6--- coefficient (1 A·s=1000 mA*1/3600 h=1/3.6 mAh)
R---pulse resistance
U_1 and I_1--- the working voltage and working current
U_2 and I_2--- the pulse voltage and pulse current

Thermal batteries differ fundamentally from conventional lithium-ion secondary batteries in working principle, electrolyte system, operating temperature, and electrode materials. The former are non-rechargeable, heat-activated devices that rely on molten salt electrolytes at high temperatures (350–550 °C), and their performance evaluation focuses on discharge behavior and pulse resistance rather than cycling life. In contrast, conventional lithium-ion batteries operate at room temperature with organic or solid-state electrolytes, and their assessment emphasizes cycling stability, rate capability, and impedance spectroscopy. These distinctions lead to very different application fields, with thermal batteries primarily used in military and aerospace applications requiring high reliability in a single discharge, while lithium-ion batteries serve widespread consumer and automotive uses. For a comprehensive comparison, Table S1 summarizes the key differences.

Table S1. Comparison: Thermal batteries vs. Conventional lithium-ion batteries

Comparison item	Thermal batteries	Conventional lithium-ion batteries
Battery type	Non-rechargeable	Rechargeable
Working principle	Activated once and discharged only	Repeated charge/discharge cycles
Cycling performance	Not applicable	Key evaluation parameter
Rate performance	Evaluated by discharge behavior under different current densities	Evaluated by charge/discharge capacities at different C-rates
Anode	High-temperature-resistant LiB alloy	Carbon
Electrolyte	Molten salt electrolyte (e.g., LiF-LiCl-LiBr)	Organic liquid electrolyte or solid electrolyte
Cathode	Transition metal sulfides, halides (FeS_2, CoS_2, NiS_2, FeF_3, $NiCl_2$, NiF_2)	$LiCoO_2$, $LiFePO_4$,
Operating temperature	350–550 °C	−20 to 60 °C

Activation method	Activated by pyrotechnic heat source to melt electrolyte	Ready for operation without activation
Electrochemical characterization	Discharge voltage/specific capacity/energy, pulse resistance	CV, EIS, cycling stability, rate capability
Internal resistance evaluation	Pulse resistance, measurement during discharge	EIS
Typical Applications	Missiles, aerospace systems, torpedoes, emergency military power supplies	Electric vehicles, portable electronics, energy storage systems
